# Effects of alfacalcidol on cardiovascular outcomes according to alkaline phosphatase levels in the J-DAVID trial

**DOI:** 10.1038/s41598-022-19820-2

**Published:** 2022-09-14

**Authors:** Tatsufumi Oka, Yusuke Sakaguchi, Yoshitaka Isaka, Haruka Ishii, Daijiro Kabata, Ayumi Shintani, Shinya Nakatani, Tomoaki Morioka, Katsuhito Mori, Masaaki Inaba, Masanori Emoto, Tetsuo Shoji

**Affiliations:** 1 Department of Nephrology, Osaka University Graduate School of Medicine, Suita, Japan; 2 Department of Inter-Organ Communication Research in Kidney Diseases, Osaka University Graduate School of Medicine, 2-2 Yamada-oka, Suita, 565-0871 Japan; 3 Medical Affairs Department, Kyowa Kirin Co., Ltd., Tokyo, Japan; 4 Department of Medical Statistics, Osaka City University Graduate School of Medicine, Osaka, Japan; 5 Department of Metabolism, Endocrinology and Molecular Medicine, Osaka City University Graduate School of Medicine, Osaka, Japan; 6 Department of Nephrology, Osaka City University Graduate School of Medicine, Osaka, Japan; 7 Vascular Science Center for Translational Research, Osaka City University Graduate School of Medicine, Osaka, Japan; 8 Department of Vascular Medicine, Osaka City University Graduate School of Medicine, Osaka, Japan

**Keywords:** Renal replacement therapy, Haemodialysis

## Abstract

In the Japan Dialysis Active Vitamin D (J-DAVID) trial, oral alfacalcidol numerically, but not significantly, increased the risk of cardiovascular events among patients undergoing hemodialysis. Because the cardiovascular effect of alfacalcidol could be modulated by bone turnover status, this post-hoc analysis of the J-DAVID examined how alkaline phosphatase (ALP), a more precise marker of bone turnover than parathyroid hormone (PTH), modifies the impact of alfacalcidol. The J-DAVID was a 48-month, open-label, randomized controlled trial comparing oral alfacalcidol with no vitamin D receptor activators use in terms of cardiovascular events among 976 hemodialysis patients without secondary hyperparathyroidism. This post-hoc analysis included 959 patients with available data on baseline ALP. The median [25–75th percentile] baseline ALP level was 234 [183–296] U/L. In a Cox proportional hazards model, ALP did not significantly modify the effect of alfacalcidol on the rate of cardiovascular events or all-cause death (*P* for effect modification = 0.54 and 0.74, respectively). The effect of alfacalcidol on time-series changes in calcium, phosphate, and intact PTH were similar across ALP subgroups. In conclusion, oral alfacalcidol did not significantly affect cardiovascular outcomes irrespective of bone turnover status.

## Introduction

Vitamin D receptor activators (VDRAs) have long been the mainstay of treatment for secondary hyperparathyroidism in patients undergoing hemodialysis^1^. In addition to its primary role in mineral and skeletal homeostasis, basic studies have shown a variety of pleiotropic effects of vitamin D, including cardiovascular (CV) protection^2^, anti-inflammatory effects^3^, immunomodulation^4^, and antitumor immunity^5^. Through these actions, VDRAs may improve the prognosis of hemodialysis patients, who have the substantial risk of CV events, infection, and malignancy^6^, independently of secondary hyperparathyroidism. Indeed, a cohort study of Italian patients undergoing hemodialysis found a significant association between VDRA use and better survival even among those with low intact parathyroid hormone (PTH) levels (≤ 150 pg/mL)^7^.

The Japan Dialysis Active Vitamin D (J-DAVID) trial was the first randomized controlled trial (RCT) to examine the efficacy of oral alfacalcidol for CV events and mortality among hemodialysis patients without overt hyperparathyroidism (intact PTH ≤ 180 pg/mL)^8^. Unexpectedly, the hazard ratio for the primary outcome, fatal and non-fatal CV events, was numerically higher in the alfacalcidol group than in the control group, although the difference was not significant (hazard ratio 1.25; 95% confidence interval, 0.94‒1.67; *P* = 0.13). This finding raised a concern that VDRAs might exert a detrimental CV effect through accelerating vascular calcification by enhancing the intestinal absorption of calcium and phosphate, especially among those with low bone turnover that impairs mineral buffering capacity^9–11^. In other words, the balance between the benefits and harms of VDRAs might depend on the status of bone turnover. Notably, PTH is a much poorer marker for low bone turnover than other markers, particularly alkaline phosphatase (ALP)^12^. Thus, patients in the J-DAVID could be heterogeneous in terms of bone turnover. In this post-hoc analysis of the J-DAVID, we explored how ALP modulates the effect of alfacalcidol on CV outcomes.

## Methods

### Ethical considerations

The J-DAVID trial was conducted in accordance with the principles of the Declaration of Helsinki. The protocol of the J-DAVID was approved by the ethics committee at the Osaka City University Graduate School of Medicine in Japan (approval numbers 1227, 1297, 1385, and 1525) and by the relevant ethics committees or institutional review boards at the study sites. All participants gave written informed consent before the study enrollment. The protocol of this post-hoc analysis was approved by the Osaka City University Graduate School of Medicine (approval number 4420).

### Study design and participants

This was a post-hoc analysis of the J-DAVID trial. Details of the J-DAVID have been described elsewhere^8^. Briefly, the J-DAVID was a 48-month, open-label, blinded endpoint randomized controlled trial that compared oral alfacalcidol with no VDRA use in terms of CV events and mortality among 976 hemodialysis patients without overt secondary hyperparathyroidism from 108 dialysis centers in Japan. The key inclusion criteria were as follows: (1) patients on maintenance hemodialysis for ≥ 90 days, (2) aged 20–80 years, (3) serum calcium levels ≤ 10.0 mg/dL, (4) serum phosphate levels ≤ 6.0 mg/dL, (5) intact PTH levels ≤ 180 pg/mL, and (6) no treatment with VDRAs within 4 weeks prior to randomization. Patients with abnormal liver function tests exceeding × 3 upper normal limits were excluded. The complete inclusion and exclusion criteria are presented in Supplementary Table 1.

Participants were randomly assigned to the oral alfacalcidol or control group in a 1:1 ratio using a computer-generated random sequence with a block randomization method. The initial dose of oral alfacalcidol was 0.5 μg/day, which was subsequently adjusted within a range of 0.25–7 μg/week to avoid hypercalcemia (serum corrected calcium levels ≥ 10.5 mg/dL), hyperphosphatemia (serum phosphate levels ≥ 7 mg/dL), and to treat hyperparathyroidism developed during the study period if these were not sufficiently managed by dietary therapy and/or dose adjustment of calcium carbonate, sevelamer hydrochloride, and other medications. Patients in the control group received usual care without alfacalcidol or any other VDRAs but were permitted to receive them if necessary (mostly when intact PTH levels exceeded their target range of the Japanese guideline^13^).

The primary outcome was fatal and non-fatal CV events, including acute myocardial infarction, coronary artery diseases requiring percutaneous coronary interventions and/or coronary artery bypass grafting, hospitalization for congestive heart failure, stroke, aortic dissection/rupture, sudden cardiac death, and endovascular treatment and/or amputation for limb ischemia. The secondary outcome was all-cause mortality. All clinical events were adjudicated independently by an event evaluation committee who was blinded to the treatment assignment. The detail definitions of CV events are presented in Supplementary Table 2.

In this post-hoc analysis, we also assessed the composite of CV events and all-cause mortality. We analyzed the time-series changes in serum corrected calcium, serum phosphate, and intact PTH levels after stratifying patients based on tertiles of baseline ALP levels. These laboratory values were measured at 0, 3, 6, 12, 18, 24, 30, 36, 42, and 48 months following randomization.

ALP was measured by the JSCC (Japanese Society of Clinical Chemistry) method in Japan when the J-DAVID trial was underway. The reference range of ALP based on the JSCC method was 100–325 U/L, which is slightly higher than that based on the IFCC (International Congress of Clinical Chemistry and Laboratory Medicine) method (38–113 U/L).

### Statistical analysis

Baseline characteristics in the alfacalcidol group and control group were summarized according to ALP tertiles. To examine whether the effect of oral alfacalcidol varied depending on the baseline ALP levels, we used multivariable Cox proportional hazard regression models considering cross-product terms between the treatment assignment and the baseline ALP levels. The models were adjusted for the baseline age, sex, body mass index, systolic blood pressure, dialysis vintage, diabetes mellitus, prior history of CV events, C-reactive protein, serum albumin, serum phosphate, serum corrected calcium, intact PTH, high-density lipoprotein cholesterol, hemoglobin, and use of erythropoiesis-stimulating agents and intravenous iron therapy. The analysis was performed using the full analysis set which consists of 964 randomized participants^8^. A potentially non-linear effect of ALP on the study outcomes was depicted using a restricted cubic spline curve with 3 knots (10th, 50th, and 90th percentiles of ALP).

To examine whether the treatment effects are modified by the baseline intact PTH rather than only the baseline ALP, we conducted additional analyses using the multivariable Cox proportional hazards regression models including three- and two-way cross-product terms between the treatment and these covariates.

Furthermore, we compared the time-series alterations in the corrected calcium, phosphate, and intact PTH levels during the observation period between the alfacalcidol and control groups using multivariable linear regression models. In these analyses, we assumed that the trends of these variables differed according to the baseline ALP levels and then divided the patients into three subgroups according to the ALP tertiles. Then, we considered three- and two-way cross-product terms between the ALP subgroup variable, month of measurement, and the treatment assignment in the multivariable linear regression models. The variance estimators of these regression models were adjusted using Huber-White method, considering the correlation between repeated measurements within a single patient. Finally, the covariates were adjusted similarly to the multivariable Cox proportional hazard regression models described above.

## Results

**Figure 1:**
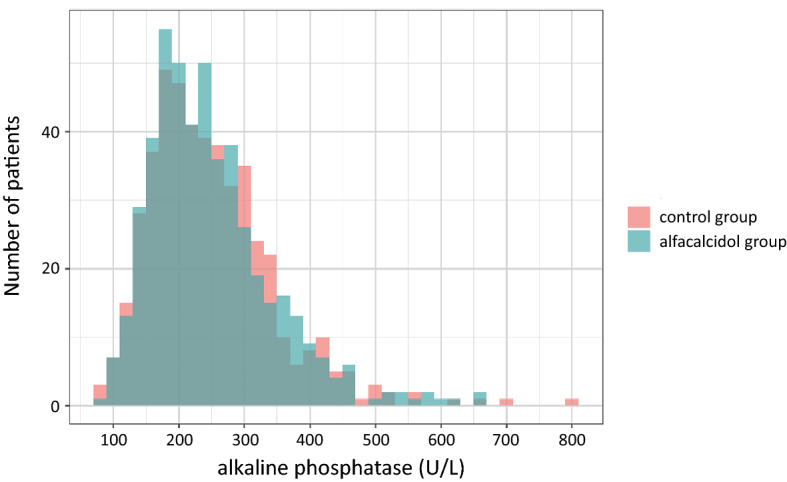
Histogram of baseline total alkaline phosphatase levels among 976 randomized patients. A total of 495 patients in the alfacalcidol group (blue) and 481 patients in the control group (red). The median [25–75th percentile] baseline alkaline phosphate level was 234 [183–296] U/L.

**Figure 2:**
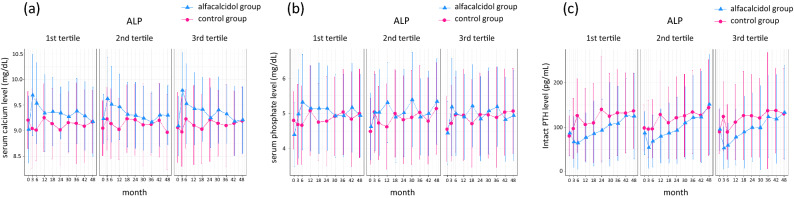
Time-series changes in laboratory data during 48-month study period stratified by alkaline phosphatase tertiles. (**a**) Serum corrected calcium level, (**b**) serum phosphate level, and (**c**) intact parathyroid hormone level. There are no significant effect modifications between the treatment assignment, alkaline phosphatase, and time for calcium (*P* = 0.21), phosphate (*P* = 0.55), and intact parathyroid hormone (*P* = 0.29).

A diagram of participant flow has been reported previously^8^. Among 964 patients in the full analysis set, 5 patients were excluded due to missing ALP data. Baseline characteristics of the study participants were similar between the alfacalcidol and control groups within each ALP strata although the mean age, the prevalence of cardiovascular comorbidities, and intact PTH levels were higher in higher ALP tertiles (Table 1). The distribution of baseline ALP levels among 959 patients is shown in Fig. 1. The median [25–75th percentile] baseline ALP was 234 [183–296] U/L. Figure 2 illustrates the time-series changes in serum corrected calcium, serum phosphate, and intact PTH levels during the 48-month study period. In the multivariable linear regression models, there were no significant coefficients of the three-way cross-product term among the treatment assignment, ALP, and time for calcium, phosphate, and intact PTH (P for effect modification = 0.21, 0.55, and 0.29, respectively), indicating that between-group differences in time-series changes in these laboratory data were not significantly altered by ALP.

**Table 1:** Baseline characteristics of study participants according to ALP tertiles.

			ALP tertiles					
			1st tertile (ALP < 196 U/L)		2nd tertile (196 U/L < = ALP < 274 U/L)		3rd tertile (274 U/L < = ALP)	
	Missing (%)	Overall	Control Group	Oral Alfacalcidol Group	Control Group	Oral Alfacalcidol Group	Control Group	Oral Alfacalcidol Group
N		964	155	165	158	161	160	160
Age (median [IQR]), y	0.0	65 [58, 71]	63 [55, 69]	62 [54, 67]	65 [59, 71]	66 [60, 70]	66 [60, 73]	68 [60, 72]
Sex, Female % (freq)	0.0	40.0 (386)	32.3 (50)	38.2 (63)	45.6 (72)	32.3 (52)	46.9 (75)	44.4 (71)
Dialysis duration (median [IQR]), y	0.0	5 [2, 11]	4 [2, 9]	5 [2, 10]	5 [2, 11]	5 [2, 10]	7 [3, 15]	6 [3, 12]
CV comorbidities, % (freq)	0.0	25.3 (244)	20.0 (31)	24.8 (41)	24.1 (38)	28.0 (45)	29.4 (47)	25.0 (40)
sBP (median [IQR]), mmHg	0.0	146 [133, 160]	147 [134, 160]	148 [133, 162]	149 [136, 162]	143 [130, 161]	148 [134, 160]	145 [129, 156]
DM % (freq)	0.0	46.0 (443)	47.7 (74)	37.6 (62)	48.7 (77)	50.9 (82)	46.2 (74)	45.6 (73)
BMI (median [IQR])	2.0	21.1 [19.1, 23.3]	21.6 [19.6, 23.4]	21.5 [19.5, 23.4]	21.0 [18.9, 23.2]	20.8 [19.1, 23.2]	20.7 [18.7, 23.1]	20.7 [18.9, 23.3]
CRP (median [IQR]), mg/dL	12.8	0.10 [0.05, 0.29]	0.09 [0.05, 0.26]	0.08 [0.05, 0.19]	0.10 [0.05, 0.23]	0.10 [0.06, 0.30]	0.11 [0.06, 0.32]	0.12 [0.06, 0.39]
ALP (median [IQR]), U/L	0.5	234 [183, 296]	164 [141, 183]	166 [146, 183]	234 [217, 255]	234 [218, 251]	328 [298, 386]	333 [295, 382]
Alb (median [IQR]), g/dL	0.1	3.8 [3.5, 4.0]	3.8 [3.6, 4.0]	3.8 [3.6, 4.0]	3.7 [3.6, 3.9]	3.8 [3.6, 4.0]	3.7 [3.5, 3.9]	3.7 [3.5, 3.9]
P (median [IQR]), mg/dL	0.0	4.7 [3.9, 5.3]	5.0 [4.2, 5.5]	4.7 [4.0, 5.4]	4.7 [3.9, 5.3]	4.5 [3.8, 5.1]	4.5 [3.8, 5.3]	4.6 [3.9, 5.1]
Corrected Ca (median [IQR]), mg/dL	0.0	9.1 [8.8, 9.5]	9.2 [8.9, 9.6]	9.2 [8.9, 9.6]	9.1 [8.7, 9.5]	9.1 [8.6, 9.4]	9.0 [8.7, 9.4]	9.1 [8.8, 9.4]
iPTH (median [IQR]), pg/mL	0.0	85 [46, 129]	78 [45, 112]	83 [46, 125]	91 [45, 128]	82 [38, 130]	97 [57, 145]	101 [51, 132]
Hb (median [IQR]), g/dL	0.0	10.6 [10.1, 11.3]	10.6 [10.0, 11.3]	10.7 [10.1, 11.3]	10.8 [10.1, 11.4]	10.6 [10.1, 11.1]	10.7 [10.1, 11.4]	10.7 [10.0, 11.5]
HDLC (median [IQR]), mg/dL	8.6	46 [37, 56]	46 [36, 55]	46 [38, 58]	47 [37, 55]	46 [37, 56]	47 [39, 56]	45 [38, 54]
ESA use, % (freq)	0.0	34.5 (333)	31.0 (48)	34.5 (57)	32.3 (51)	31.7 (51)	38.1 (61)	38.1 (61)

*ALP* alkaline phosphatase; *CV* cardiovascular disease; *sBP* systolic blood pressure; *DM* diabetes mellitus; *BMI* body mass index, *CRP* C-reactive protein, *Alb* serum albumin, *P* serum phosphate, *Corrected Ca* albumin-corrected serum calcium, *iPTH* intact parathyroid hormone, *Hb* hemoglobin, *HDLC* high-density lipoprotein cholesterol, *ESA* erythropoiesis-stimulating agents, *IQR* interquartile range.

**Figure 3:**
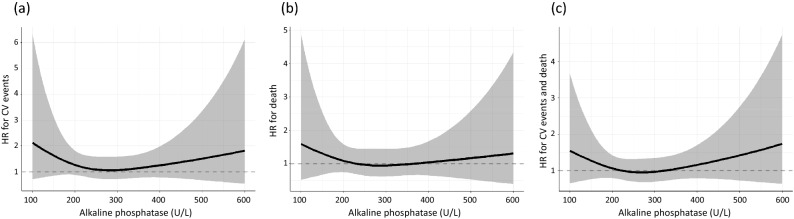
Alkaline phosphatase does not modify the effect of alfacalcidol on the cardiovascular outcomes and mortality. Cubic spline curves for the hazard ratios of the alfacalcidol groups vs. the control group in terms of (**a**) cardiovascular events, (**b**) all-cause death, and (**c**) composite of cardiovascular events and all-cause death are depicted with 3 knots at 10th, 50th, and 90th percentiles of ALP. The Cox models were adjusted for age, sex, body mass index, systolic blood pressure, dialysis vintage, diabetes mellitus, prior history of cardiovascular events, C-reactive protein, serum albumin, serum phosphate, serum corrected calcium, intact parathyroid hormone, high-density lipoprotein cholesterol, hemoglobin, and use of erythropoiesis-stimulating agents and intravenous iron therapy. The lines and gray zones indicate the hazard ratios and 95% confidence intervals. *P*-values for the coefficient of the cross-product term between the treatment assignment and alkaline phosphatase were 0.54, 0.74, and 0.50 for cardiovascular events, all-cause death, and composite of cardiovascular events or all-cause death.

The multivariable Cox proportional hazard regression models showed that the cross-product term between the treatment assignment and ALP was not significant with respect to the rate of CV events (P for effect modification = 0.54) (Fig. 3). Similar results were obtained for the rate of all-cause death and the composite of CV events and all-cause death (P for effect modification = 0.74 and 0.50, respectively) (Fig. 3). In the additional analysis, the hazard ratios for the study outcomes in the alfacalcidol group compared to the control group were not significant when both ALP and intact PTH were low and when both ALP and intact PTH were high (Table 2).

**Table 2:** Hazard ratios for cardiovascular events and deaths in the alfacalcidol group based on both ALP and PTH.

Subgroups		Fatal and non-fatal CV events		All-cause mortality		CV events and mortality	
iPTH	ALP	HR* [95% CI]	*P*-value	HR* [95% CI]	*P*-value	HR* [95% CI]	*P*-value
46 pg/mL	183 U/L	1.54 [0.91–2.60]	0.11	0.99 [0.58–1.70]	0.98	1.14 [0.75–1.72]	0.54
	234 U/L	1.33 [0.82–2.16]	0.25	0.89 [0.53–1.49]	0.65	1.03 [0.68–1.55]	0.90
	296 U/L	1.18 [0.69–2.01]	0.54	0.90 [0.51–1.59]	0.71	1.00 [0.64–1.57]	0.99
85 pg/mL	183 U/L	1.38 [0.90–2.11]	0.14	1.20 [0.76–1.88]	0.44	1.15 [0.82– 1.62]	0.43
	234 U/L	1.16 [0.80–1.66]	0.44	0.99 [0.67–1.45]	0.94	0.99 [0.73–1.35]	0.97
	296 U/L	1.08 [0.72–1.61]	0.71	0.93 [0.61–1.43]	0.74	0.97 [0.69–1.35]	0.85
129 pg/mL	183 U/L	1.22 [0.66– 2.27]	0.52	1.47 [0.75–2.89]	0.26	1.16 [0.70–1.92]	0.56
	234 U/L	0.99 [0.62–1.57]	0.96	1.11 [0.68–1.82]	0.67	0.96 [0.66–1.40]	0.82
	296 U/L	0.98 [0.59–1.63]	0.93	0.97 [0.56–1.68]	0.91	0.94 [0.62–1.42]	0.76

*iPTH* intact parathyroid hormone, *ALP* alkaline phosphatase, *HR* hazard ratio, *CI* confidence interval.

*HR in the alfacalcidol group vs the control group at each specified iPTH and ALP values.

## Discussion

In this post-hoc analysis of the J-DAVID, we found that the effect of alfacalcidol on the risk of CV events, all-cause deaths, or their composite was not significantly modified by ALP among hemodialysis patients with baseline intact PTH levels ≤ 180 pg/mL. Similarly, the effects of alfacalcidol on time-series changes in calcium, phosphate, and intact PTH levels were comparable across the baseline ALP categories.

While observational studies have reported an association between the use of VDRAs and better prognosis in hemodialysis patients^14,15^, the J-DAVID showed a numerically higher risk of CV events in the alfacalcidol group^8^. This may be owing to a harmful aspect of VDRAs, which might outweigh the benefits of these drugs. When used for patients without hyperparathyroidism, VDRAs could further suppress bone turnover and impair mineral buffering capacity. As a result, elevated calcium and phosphate loads due to enhanced intestinal absorption caused by VDRAs might accelerate vascular calcification^9–11^. To address this issue in more detail, we used ALP to better specify the patients’ bone turnover status. Indeed, the area under the receiver operating characteristic curve (AUC) of the total ALP for discriminating low bone turnover in patients with advanced chronic kidney disease (CKD) is reported to be 0.753, as compared to 0.606 for intact PTH^12^. Given the poor discrimination ability of PTH, participants in the J-DAVID are expected to have various bone turnover status. With a more precise assessment of bone turnover by ALP, we sought to determine how the effect of alfacalcidol was modified by bone turnover.

Contrary to our hypothesis, ALP did not significantly modify the effect of alfacalcidol on CV outcomes. There are two potential explanations for this result. First, the dose of alfacalcidol (initial dose: 0.5 μg/day) might not be enough to modulate the bone-vascular axis. This is suggested by the fact that between-group differences in calcium, phosphate, and PTH levels during the study period were small and not modified by ALP. Although PTH levels were lower in the alfacalcidol group than in the control group throughout the study period, the difference between the two groups became gradually smaller as the study progressed probably due to the discontinuation of alfacalcidol in the alfacalcidol group in addition to the initiation of VDRAs in the control group. In the ADVANCE study, which compared VDRAs alone with a combination of cinacalcet and low-dose VDRAs, the former showed greater progression of coronary and valvular calcifications accompanied by remarkable between-group differences in calcium and phosphate; patients in the VDRAs group received 12.6 μg/week of paricalcitol on average, which is equivalent to nearly 1 μg/day of oral alfacalcidol^16^. Thus, when used at low doses as in the J-DAVID, the potential harm of VDRAs on CV outcomes may not manifest even among those with low bone turnover.

Second, it has not been formally verified whether low bone turnover per se actually predisposes to the progression of vascular calcification and augments CV risks. Although observational studies reported associations between low bone turnover and vascular calcification, they did not prove causation^9–11^. In a cohort of incident hemodialysis patients, naturally decreasing PTH levels were associated with a much higher risk of mortality than treatment-induced low PTH levels^17^, suggesting that factors contributing to low bone turnover, such as diabetes mellitus, malnutrition, and inflammation, are more directly involved in CV risks^18^. It is also unclear to what extent calcium load affects the risk of CV events^19^. In a randomized trial of 2,309 patients undergoing hemodialysis, no significant differences were found between calcium carbonate and lanthanum carbonate in terms of the CV events^20^. Notably, the median intact PTH of this trial (107.2–114.0 pg/mL) was similar to that of the J-DAVID (85.1–86.1 pg/mL). Our results suggest that low-dose VDRAs do not aggravate CV risks even among those with pre-existing low bone turnover defined by low ALP.

The Kidney Disease Outcome Quality Initiative guideline recommends withholding VDRAs if intact PTH levels are less than 150 pg/mL^21^. Our current analysis did not show a significant increase in CV risk and mortality in the alfacalcidol group even when bone turnover markers were suppressed. It must be recognized, however, that the statistical power of our subgroup analyses may not be enough to detect a significant between-group difference. In fact, there was a trend toward worse prognosis in the alfacalcidol group. Thus, caution must be taken when prescribing VDRAs for those with low bone turnover. Adequately powered trials are required to conclude the impact of VDRAs in such patients.

Alfacalcidol did not exhibit a favorable effect on CV outcomes even among patients without low ALP who were likely to have normal or high bone turnover. Although animal studies have reported CV protective effects of VDRAs^22,23^, previous RCTs in non-dialysis CKD found no beneficial effects of paricalcitol on left ventricular structure and function^24,25^. A recent meta-analysis has shown a neutral effect of vitamin D supplementation on CV outcomes in both CKD and non-CKD populations^26^. Compared to alfacalcidol, calcimimetics have reduced left ventricular mass among hemodialysis patients, which was correlated with a decrease in fibroblast growth factor 23^27^. Calcimimetics have also been more effective to attenuate serum calcification propensity, T50, than maxacalcitol^28^. In addition, calcimimetics would be preferred to achieve simultaneous control of PTH and phosphate; strict phosphate control has been shown to attenuate the progression of coronary artery calcification^29^. Taken together, calcimimetics may have an advantage over VDRAs to mitigate CV risks among hemodialysis patients.

The present study had some limitations. First, this was a post-hoc*,* not pre-planned, analysis of the J-DAVID trial. The study protocol was not specifically designed to test the cross-product between the treatment assignment and ALP. Thus, the statistical power to detect significant effect modifications might be limited. Larger studies are warranted to confirm whether VDRAs neither improve nor worsen the prognosis of hemodialysis patients. Second, because randomization was not stratified by ALP, there could be potential differences in the measured and unmeasured covariates between the study groups in each ALP strata, although extensive adjustment for baseline characteristics in the multivariate models was performed to address this issue. Third, some participants in the control group received VDRAs during the study period, whereas some in the alfacalcidol group stopped the drug^8^. This treatment contamination might have diluted the true intervention effect of alfacalcidol. Fourth, we used total ALP, but not bone-specific ALP. Total ALP could be affected by factors other than bone formation, such as liver diseases although the J-DAVID trial excluded patients with abnormal liver function tests. Fifth, we did not take into account ALP after administration of alfacalcidol. We believe, however, that pre-treatment values are particularly of importance in deciding the initiation of the drug. Our study was not designed to assess how post-intervention ALP levels influence the effect of alfacalcidol on cardiovascular outcomes, which should be investigated by future studies. Sixth, we did not have information about bone histology which is the gold standard for the evaluation of bone turnover. Finally, our study was limited to J-DAVID participants, thereby limiting the generalizability to those with overt secondary hyperparathyroidism.

In conclusion, the effect of oral alfacalcidol on the risk of CV events and mortality was not substantially modified by ALP among hemodialysis patients without overt secondary hyperparathyroidism. The present results suggest that low-dose alfacalcidol does not worsen the prognosis of patients with pre-existing low bone turnover, but is unlikely to improve CV outcomes irrespective of the bone turnover status.

## Supplementary Information

Supplementary Information.

## Data Availability

The data underlying this article cannot be shared publicly due to the privacy of individuals that participated in the study. The data will be shared on reasonable request to the corresponding author.
