# Supplementary material for: Effects of alfacalcidol on cardiovascular outcomes according to alkaline phosphatase levels in the J-DAVID trial
Source: Sci Rep. 2022 Sep 14;12:15463. doi: 10.1038/s41598-022-19820-2 (PMC9475027; doi:10.1038/s41598-022-19820-2)
Supplement: Supplementary file 1 — Supplementary Information. [file 41598_2022_19820_MOESM1_ESM.docx]

**Supplemental Material**

**Table of Contents**

Supplementary Table 1: Complete inclusion and exclusion criteria of the J-DAVID trial

Supplementary Table 2: Definitions of cardiovascular events

**Supplementary Table 1: Complete inclusion and exclusion criteria of the J-DAVID trial**

| **Inclusion criteria** |
| --- |
| 1. Signed informed consent  2. Patients on maintenance hemodialysis for 90 days or longer  3. Men or women aged >20 and <80 years old  4. No treatment with VDRAs for more than 4 weeks prior to this study  5. Serum calcium level <10.0 mg/dL  6. Serum phosphate level <6.0 mg/dL  7. Serum intact PTH level <180 pg/mL |
| **Exclusion criteria** |
| 1. History within 12 weeks of myocardial infarction, stroke, aortic dissection/rupture, amputation of a lower limb, coronary revascularization or bypass surgery, lower limb revascularization or bypass surgery  2. Heart failure of NYHA grade III or IV  3. Respiratory failure with PaO2 <60 mmHg or SpO2<90%  4. Life expectancy shorter than 1 year due to known malignant, infectious, or other diseases  5. Abnormal liver function tests exceeding x3 upper normal limits  6. Pregnant or lactating females or females planning to be pregnant  7. History of an allergic reaction to alfacalcidol  8. Participation to other interventional studies within 12 weeks prior to this study  9. Inappropriate for this study as judged by an attending investigator |

| **Supplementary Table 2: Definitions of cardiovascular events**  **Acute myocardial infarction:**  Clinical signs and symptoms such as chest pain or cardiogenic shock, associated with abnormalities in biomarkers (creatine kinase, troponin, etc.) and/or electrocardiogram (new abnormal Q-wave, ST elevation, etc.) for myocardial infarction. |
| --- |
| **Congestive heart failure:**  Congestive heart failure (NYHA grade III or IV) requiring hospitalization, excluding dyspnea due to non-cardiac causes (bronchial asthma, etc.) |
| **Stroke:**  Rapidly developing clinical signs of neurological deficit attributable to a focal and/or total brain functions, without clear causes than vascular origin, lasting for more than 24 hours or leading to death (if not interrupted by surgical operations or death). Stroke includes subarachnoidal hemorrhage, intracranial hemorrhage, and cerebral infarction, but excludes transient ischemic attack, cerebrovascular disease due to hematological disorders (leukemia, polycytemia vera, etc.), primary brain tumors, and metastatic brain tumors. Stroke secondary to trauma is also excluded. |
| **Aortic dissection/rupture:**  Clinical symptom of chest pain and/or abdominal pain, and diagnosed with imaging test such as contrast enhanced computed tomography. |
| **Amputation of ischemic limb:**  Major amputations at ankle joint or proximal as treatment for patients with symptom and/or signs of lower extremity ischemia. |
| **Cardiac sudden death:**  Unexpected death from a cardiac cause that occurs within one hour of symptom onset (witnessed) or within 24 hours of last being observed in normal health (unwitnessed). |
